# Abcès tuberculeux de la paroi thoracique chez l'enfant

**DOI:** 10.11604/pamj.2013.14.9.2138

**Published:** 2013-01-06

**Authors:** Rachid El Barni, Mohamed Lahkim, Abdessamad Achour

**Affiliations:** 1 Service de chirurgie générale, Hôpital militaire Avicenne, Marrakech, Maroc

**Keywords:** Tuberculose, paroi thoracique, abcès, enfant, Tuberculosis, chest wall, abscess, child

## Abstract

La localisation pariétale thoracique chez l'enfant est une forme très rare de tuberculose. Son diagnostic est souvent difficile nécessitant le recours à la biopsie chirurgicale. Nous rapportons le cas d'une jeune fille de 14 ans, présentant depuis un mois et demi une tuméfaction de la paroi thoracique antérieure. La tomodensitométrie thoracique a objectivé un processus lésionnel pariétal para-sternal droit à limites imprécises responsable d'une érosion du sternum. L'examen anatomo-pathologique des biopsies de la coque d'abcès a confirmé le diagnostic de tuberculose caséo-folliculaire. Le traitement anti-bacillaire a permis une évolution favorable.

## Introduction

La tuberculose redevient une maladie d'actualité et peut revêtir des formes cliniques trompeuses et intéresser des localisations inhabituelles. La localisation à la paroi thoracique est rare. Il s'agit d'une présentation inhabituelle de la tuberculose extra-pulmonaire, et représente moins de 5% des atteintes tuberculeuses ostéo-articulaires, évaluées elles-mêmes à 15% des tuberculeuses extra-pulmonaires [1]. Elle concerne exceptionnellement l'enfant, même en zone de forte endémie [2–5]. Le diagnostic repose sur l'analyse anatomopathologique des tissus et/ou des prélèvements bactériologiques. Nous rapportons un cas de tuberculose pariétale thoracique chez une patiente immuno-compétente de 14 ans avec une revue de la littérature.

## Patient et observation

**Figure 1:**
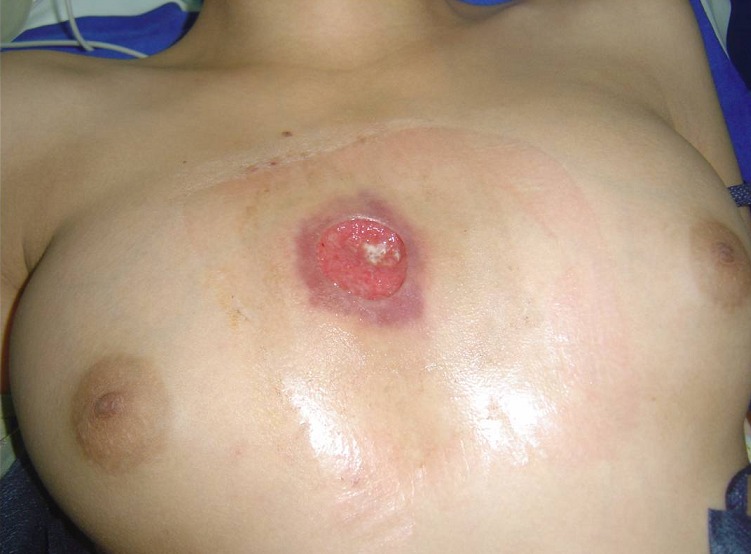
Aspect après mise à plat d'un abcès de la paroi thoracique antérieure

**Figure 2:**
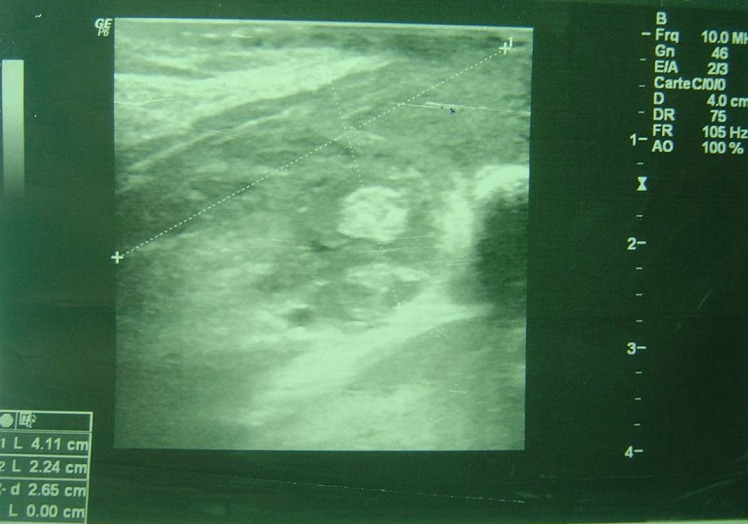
Echographie des parties molles du thorax: collection hypoéchogène hétérogène à limites irrégulières au niveau de la paroi thoracique antérieure et en sous-cutané mesurant 41mm/22mm associée à des remaniements ostéolytiques du sternum

**Figure 3:**
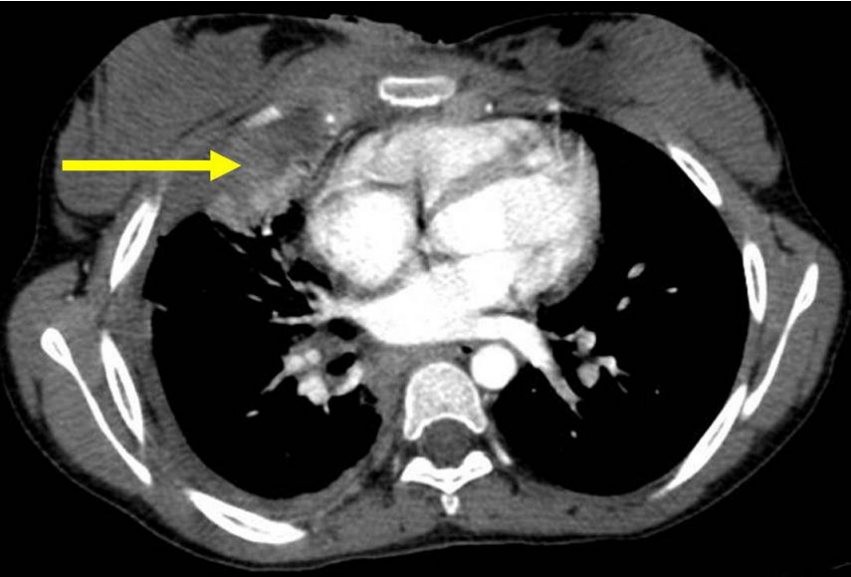
Tomodensitométrie thoracique: processus lésionnel pariétal para-sternal droit à limites imprécises mesurant 51mm/32mm, responsable d'une érosion du sternum et se continuant avec un foyer de condensation parenchymateuse du segment interne du lobe pulmonaire moyen

**Figure 4:**
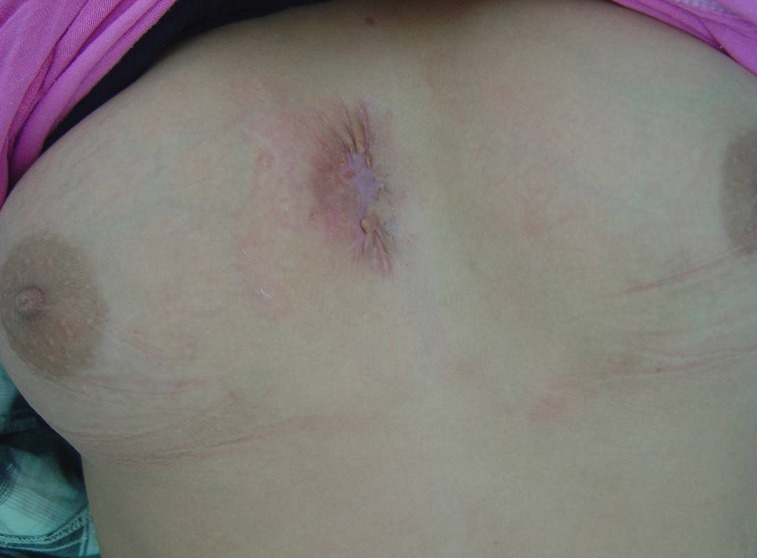
Aspect cicatriciel de la lésion pariétale thoracique après 3 mois de traitement anti-bacillaire

TC enfant de 14 ans, vaccinée contre la tuberculose, présente une tuméfaction para-sternale droite indolore évoluant depuis un mois et demi. L'examen physique trouve une tuméfaction para-sternale droite, mesurant 4 cm de grand axe, fluctuante en son centre, avec une peau inflammatoire en regard (Figure 1). Le reste de l'examen somatique met en évidence des adénopathies axillaires bilatérales. Le bilan biologique trouve une hyperleucocytose à 12500/mm^3^ et une vitesse de sédimentation à 50 mm à la 1ère heure. La sérologie du virus de l'immuno-déficience humaine et le bilan phtysiologique sont négatifs. L’échographie thoracique montre une collection hypoéchogène hétérogène à limites irrégulières au niveau de la paroi thoracique antérieure et en sous-cutané mesurant 41mm/22mm associée à des remaniements ostéolytiques du sternum (Figure 2). La tomodensitométrie thoracique objective un processus lésionnel pariétal para-sternal droit à limites imprécises mesurant 51mm/32mm discrètement rehaussé après injection du produit de contraste, responsable d'une érosion du sternum et semblant être en continuité avec un foyer de condensation parenchymateuse du segment interne du lobe pulmonaire moyen (Figure 3), ceci est associé à des adénopathies médiastino-axillaires. Nous avons réalisé une biopsie ganglionnaire axillaire, une mise à plat de la masse abcédée avec biopsies des berges, un prélèvement de pus pour étude bactériologique et recherche du bacille de Koch qui revient négative après culture sur milieu solide de Löwenstein-Jensen. L'examen anatomo-pathologique des biopsies note un aspect évocateur de tuberculose caséo-folliculaire avec adénite réactionnelle. La patiente est traitée par les antibacillaires pendant neuf mois. L’évolution est favorable après trois mois de traitement avec une cicatrisation de la lésion pariétale thoracique (Figure 4).

## Discussion

La tuberculose de la paroi thoracique est rare. Sa fréquence est de 1 à 5% des localisations ostéo-articulaires et de 0,1% de toutes les formes de tuberculose [1]. Les abcès tuberculeux de la paroi thoracique sont rares. L'atteinte pariétale est due souvent à un drainage lymphatique à travers une plèvre infectée, ou bien par contiguïté quand un empyème tuberculeux se rompt dans les parties molles. Ces masses pariétales peuvent parfois faire discuter une origine tumorale [6]. La tuberculose de la paroi thoracique peut être isolée ou associée à une localisation pulmonaire ou médiastinale, voire multifocale [2, 4]. Les antécédents de tuberculose sont rencontrés en cas d'abcès froid de la paroi thoracique chez 83% des patients [7], et une tuberculose active est concomitante dans 17,4% à 62,5% des cas [8]. L'abcès froid se localise assez souvent au niveau de la paroi antérolatérale du thorax [9, 10]. La masse est rarement fluctuante, ce qui ne fait évoquer son origine infectieuse que rarement [1]. L'empyème de nécessité d'origine tuberculeuse est caractérisé par l'absence de signes inflammatoires. Une douleur de type pleurale, une toux sèche, une fièvre modérée, des sueurs nocturnes et un amaigrissement peuvent être présents ou faire défaut. Les symptômes peuvent être absents s'il existe une fistule broncho-pleurale ou un empyème de nécessité [6].

La radiographie du thorax révèle souvent un épanchement pleural ou un épaississement pleural, ou une calcification pleurale. Ailleurs, une opacité pariétale peut être visualisée [6]. Quant à l’échographie, elle peut montrer le caractère ramolli de la masse et en guider la biopsie [1].

La tomodensitométrie thoracique montre des anomalies peu caractéristiques, en dehors de l'empyème de nécessité [1]. Elle peut même égarer le diagnostic vers une origine tumorale [1]. En cas d'abcès froid secondaire à un empyème de nécessité, la tomodensitométrie peut faire découvrir une masse pleurale bien encapsulée, fistulisée à la paroi thoracique ou abdominale; la fistule est souvent non détectée du fait de sa petite taille [6]. Ces examens permettent de faire le bilan lésionnel et de réaliser une ponction-biopsie trans-pariétale pour études bactériologiques et/ou histologiques [10].

Le diagnostic de certitude de l'origine tuberculeuse repose sur l'isolement de *Mycobacterium tuberculosis* dans le liquide de ponction et/ou dans les fragments de biopsies ou sur l’étude histologique des biopsies ou des pièces d'exérèses chirurgicales [8, 10]. Le recours à la technique polymerase chain reaction peut être d′un grand apport et poser rapidement le diagnostic afin de débuter un traitement antituberculeux précocement [7]. Le traitement de l'abcès froid est controversé. L'association de la chirurgie au traitement antituberculeux constitue la seule garantie de guérison définitive [1], et elle est préférentiellement préconisée afin de réduire les récidives [6]. Le traitement médicamenteux classique de la tuberculose repose sur une quadrithérapie antituberculeuse de deux mois (isoniazide, rifampicine, éthambutol et pyrazinamide) suivie d'une bithérapie (isoniazide et rifampicine), avec une durée totale de traitement de neuf à 12 mois [1, 7, 8]. Ce traitement médical est précédé d'un traitement chirurgical permettant d’évacuer voire même réséquer l'abcès en totalité et d'emporter les tissus nécrosés sous-jacents [7]. Le pronostic est le plus souvent favorable, bien qu'il dépende du délai diagnostique et de la rapidité de la mise en route du traitement [1, 8].

## Conclusion

En l'absence d'autres lésions pulmonaires ou extra-pulmonaires évocatrices de tuberculose, il est parfois difficile de distinguer un abcès froid tuberculeux d'une tumeur de la paroi thoracique. La symptomatologie clinique est peu spécifique. L'imagerie et, en particulier, l’échographie et la tomodensitométrie thoracique peuvent parfois aider au diagnostic. La biopsie chirurgicale et de la preuve histologique sont nécessaires afin d’éliminer une origine néoplasique particulièrement chez les patients immunocompétents. Le pronostic est habituellement bon sous polychimiothérapie tuberculeuse associée à une exérèse chirurgicale complète de l'abcès.
